# Painting Cell–Cell Interactions by Horseradish Peroxidase and Endogenously Generated Hydrogen Peroxide

**DOI:** 10.1021/acschembio.4c00419

**Published:** 2024-12-18

**Authors:** Youngjoon Cho, Inyoung Jeong, Kwang-eun Kim, Hyun-Woo Rhee

**Affiliations:** 1 Department of Chemistry, 26725 Seoul National University, Seoul 08826, Korea; 2 Department of Convergence Medicine, 37974 Yonsei University Wonju College of Medicine, Wonju 26426, Korea; 3 School of Biological Sciences, 26725 Seoul National University, Seoul 08826, Korea

## Abstract

Cell–cell interactions are fundamental in biology for maintaining physiological conditions with direct contact being the most straightforward mode of interaction. Recent advancements have led to the development of various chemical tools for detecting or identifying these interactions. However, the use of exogenous cues, such as toxic reagents, bulky probes, and light irradiation, can disrupt normal cell physiology. For example, the toxicity of hydrogen peroxide (H_2_O_2_) limits the applications of peroxidases in the proximity labeling field. In this study, we aimed to address this limitation by demonstrating that membrane-localized horseradish peroxidase (HRP-TM) efficiently utilizes endogenously generated extracellular H_2_O_2_. By harnessing endogenous H_2_O_2_, we observed that HRP-TM-expressing cells can effectively label contacting cells without the need for exogenous H_2_O_2_ treatment. Furthermore, we confirmed that HRP-TM labels proximal cells in an interaction-dependent manner. These findings offer a novel approach for studying cell–cell interactions under more physiological conditions without the confounding effects of exogenous stimuli. Our study contributes to elucidating cell–cell interaction networks in various model organisms, providing valuable insights into the dynamic interplay between cells in their native network.

## Graphical abstract

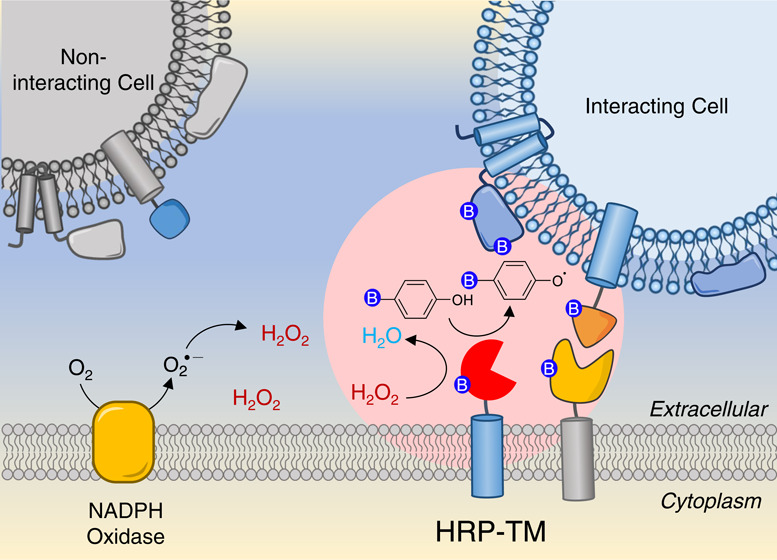

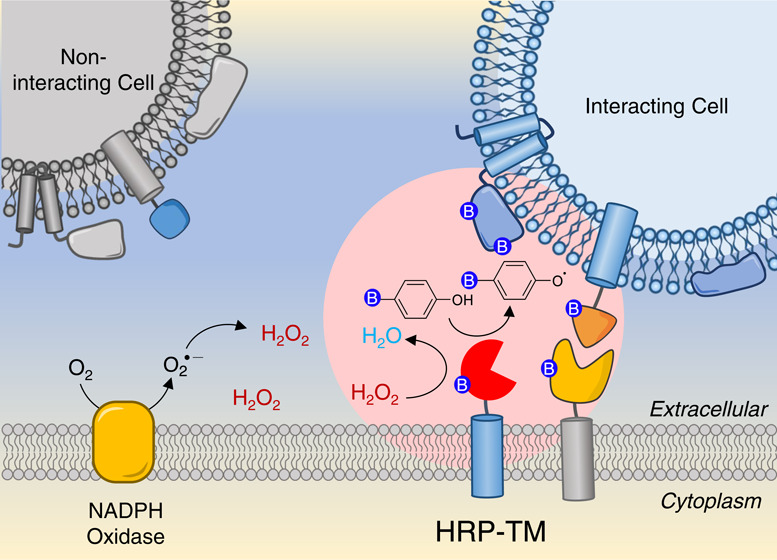

Cell–cell interactions are crucial for regulating cellular physiology, playing essential roles in neuronal signaling, tissue formation, immune responses, and tumor progression.^1–4^ T cells, for instance, are activated when they recognize antigen-presenting cells by direct contact, while tumor microenvironments consist of interactions among various cell types, including cancer cells and noncancerous cells such as immune cells, endothelial cells, or fibroblasts.^5^ It is essential to identify intercellular networks at the tissue level, while maintaining physiological conditions.

Recently, several studies on cell–cell interactions have been reported with diverse approaches. One of the most widely used is a fluorescence-based method. Porterfield et al. developed a tool to detect cell–cell contact via luciferase–luciferin reaction.^6^ In addition, Tang et al. suggested a new tool, G-baToN, in which GFP and anti-GFP nanobody (αGFP) record the intercellular interactions.^7^ Another method based on a cell-penetrating fluorescent protein was suggested by Ombrato et al., as a system for identifying the spatial cellular environment of metastatic cancers.^8^ However, the above methods require engineered ligand and receptor expression, which makes it impossible to reveal cell–cell interactions between unknown cell types, and the interaction between an artificial ligand–receptor pair could influence physiological cell–cell contact events.

Proximity labeling has become a versatile tool for studying spatiotemporal proteomics and has emerged as a powerful tool for deciphering cell–cell interaction.^1^ LIPSTIC, which utilizes sortaseA (SrtA) to transfer LPXTG-biotin to N-terminal oligoglycine of proteins at the cell surface, confirmed the interactions of dendritic cells and T cells.^9^ Recently, it has been developed into EXCELL,^10^ an engineered SrtA (mgSrtA) to functionalize the enzyme for transferring LPXTG-biotin to N-terminal monoglycine. However, the relatively bulky conjugate (LPETG = 520 Da) compared to biotin (244 Da) makes the tissue penetration of LPETG-biotin uncertain. PUP-IT^11^ and FucoID^12,13^ have also been reported as methods that allow exogenous enzymes to be expressed in bait cells, enabling protein/peptide or glycosylation probes to be displayed on prey cells. In this case, direct cell–cell interactions can be selectively detected by enzymatic reaction; however, efficient delivery of large molecular weight probes (e.g., LPETG peptide, PUP protein, fucosylation donor) for these proximity labeling methods should be optimized for *in vivo* application.

Recently, photocatalysts have been developed to capture spatial biological network or spatiome.^14^ MicroMap (μMap), a photocatalytic reaction of iridium that generates reactive carbene species upon blue-light irradiation, has been suggested as a tool with a significantly short labeling radius of around 4 nm^15^. Oslund et al. have designed riboflavin tetraacetate (RFT)-mediated labeling of proteins, called photocatalytic cell-tagging (PhoTag).^16^ Liu et al. reported antigen-specific T cell detection using the photosensitizer dibromofluorescein (DBF) termed PhoXCELL,^17^ and Qiu et al. developed a Ru-^1^O_2_-hydrazide system for photocatalytic cell labeling.^18^ However, light irradiation for photocatalytic reactions critically limits their use to *in vitro* or cell-level applications rather than *in vivo*.

Owing to the aforementioned potential limitations of proximity labeling tools for *in vivo* application for labeling cell–cell interaction, we hypothesized that peroxidases could effectively address these challenges. Peroxidases such as APEX2 and horseradish peroxidase (HRP) catalyze the oxidation of phenol substrates like desthiobiotin-phenol (DBP = 333.43 Da)^19^ to generate phenoxy radicals in the presence of hydrogen peroxide (H_2_O_2_). This reaction enables protein labeling by forming covalent bonds between biotin-phenoxy radicals and electron-rich amino acid residues such as tyrosine. Previous studies have utilized exogenous HRP, split HRP, or nucleic acid–based HRP mimics, such as G-quadruplex/hemin, to label and identify surface proteomes and intercellular protein–protein interactions in various *in vitro* or *ex vivo* models.^20–23^ However, the toxicity of exogenous H_2_O_2_ treatment limits its application.

Recent research has demonstrated that live cells generate endogenous H_2_O_2_ under physiological conditions.^24–26^ In the extracellular space, endogenous reactive oxygen species (ROS) is generated by NADPH oxidase (NOX)^27–30^ or H_2_O_2_ can also diffuse directly across the membrane via aquaporin.^31^ Consequently, extracellular H_2_O_2_ is continuously produced through this process. A study by Zhu et al. has reported that endogenously generated H_2_O_2_ can activate a chemical probe for proximal protein labeling,^24^ and our group also reported that subcellularly expressed APEX2 can be activated by endogenously generated ROS in live cells.^25^ Wang and colleagues successfully labeled protein on the cell surface using HRP, utilizing endogenously generated H_2_O_2_ during cell starvation.^26^ Thus, we hypothesized that the APEX2/HRP reaction could utilize H_2_O_2_ on the cell contact under physiological conditions.

To develop a proximity labeling tool for mapping intercellular interactions, we designed an APEX2/HRP construct with an Igκ leader signal peptide (SP) in the N-terminus and the transmembrane domain (TM) of PDGF receptor beta, which exposes the peroxidase extracellularly. We anticipated that the peroxidase reaction with DBP, with or without H_2_O_2_ treatment, would biotinylate surface proteins of HRP-TM-expressing cells and their proximal cells (Figure 1).

**Figure 1:**
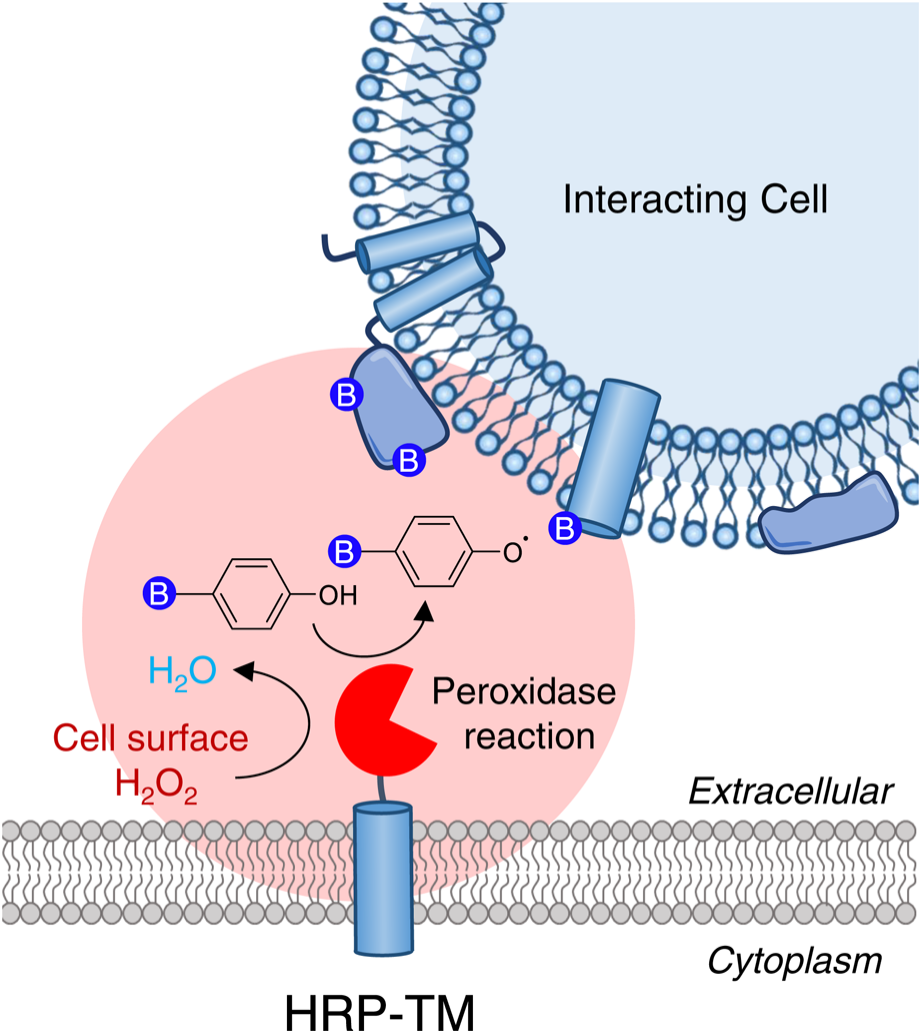
Scheme of endogenous hydrogen peroxide-assisted proximal cell labeling. HRP is expressed in the extracellular space by fusing the transmembrane domain (TM). Upon biotin-phenol addition, HRP biotinylates membrane proteins of proximal cells by utilizing endogenous H_2_O_2_ generated in the extracellular space.

## Results

First, we compared the utilization of endogenous H_2_O_2_ between APEX2 and HRP. HRP-TM or APEX2-TM was transiently expressed in HEK293T cells to compare the labeling efficiency with or without exogenous H_2_O_2_ treatment. HRP-TM is fused with an Myc tag, and APEX2-TM is fused with a V5 tag, respectively. Anti-Myc and Anti-V5 blots confirmed the expression of each construct (Figure 2A). Nonspecific bands in the V5 blot indicated equal protein loading. Biotinylated proteins were detected via the streptavidin (SA)–HRP blot. With additional H_2_O_2_ treatment, both HRP and APEX2 effectively biotinylated proteins (Figure 2A). Immunofluorescence imaging confirmed the labeling of the cell surface under H_2_O_2_ treatment condition (Figure S1).

**Figure 2:**
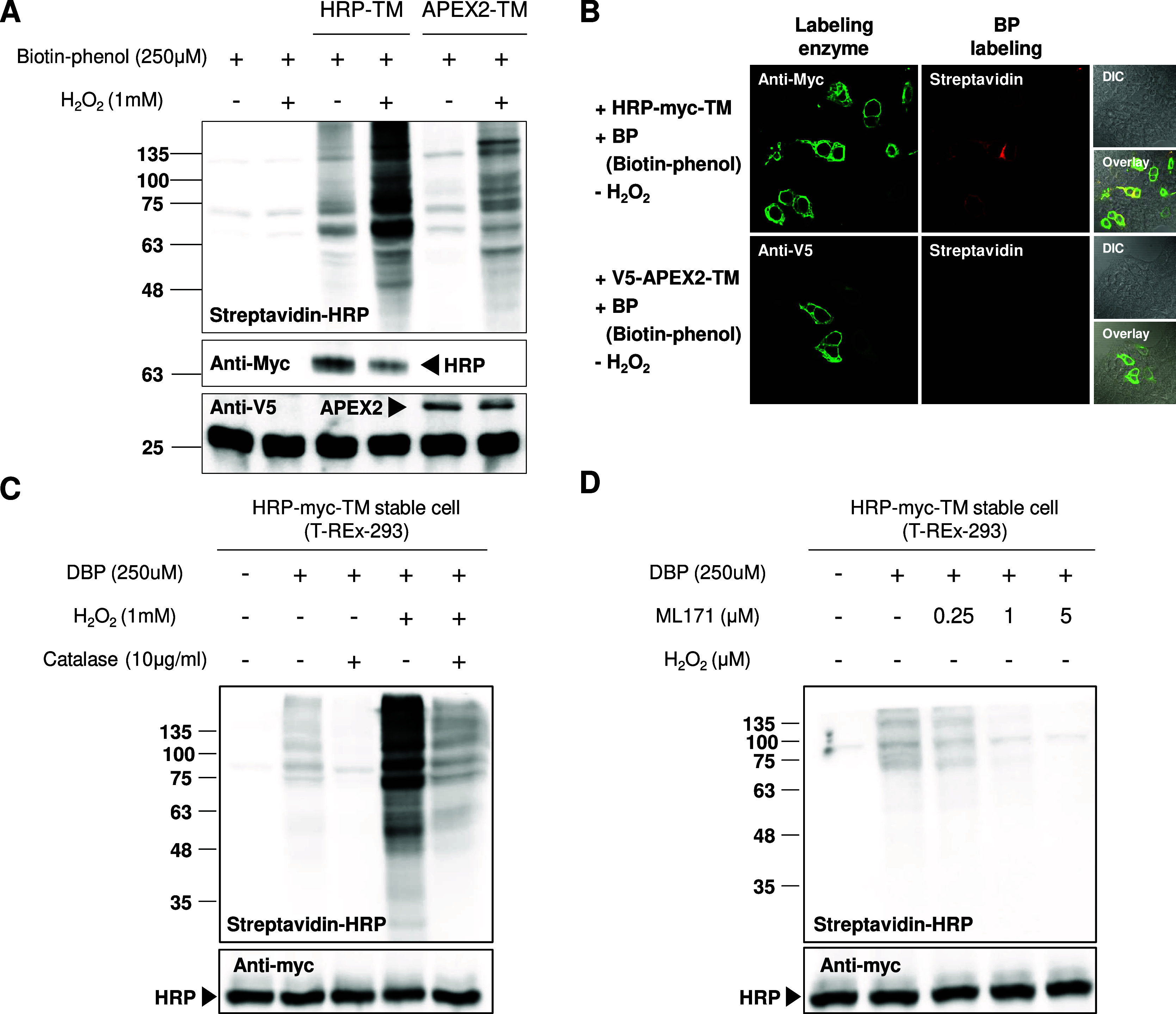
Comparison of H_2_O_2_-facilitated peroxidase activity on the cell surface and endogenous H_2_O_2_-induced cell surface labeling via NOX. (A) Western blot analysis of biotinylated proteins (streptavidin-HRP, SA-HRP) labeled by HRP-TM or APEX2-TM with or without exogenous H_2_O_2_ treatment. HEK293T cells were treated with 250 μM biotin-phenol (BP) for 30 min at 37 °C, followed by 1 mM H_2_O_2_ for 1 min at RT. (B) Immunofluorescence imaging of HRP-TM (Anti-Myc) or APEX2-TM (Anti-V5) and biotinylated proteins (streptavidin-Alexa) in HEK293T cells. (C) Inhibition of HRP-TM labeling by catalase treatment. HRP-myc-TM stable cells were incubated with DBP (250 μM) and catalase (10 μg/mL) for 30 min simultaneously, followed by 1 mM H_2_O_2_ for 1 min. (D) Western blot data showing the effect of the NOX inhibitor ML171 on labeling. HRP-TM stable cells were pretreated with ML171 for 1 h at 37 °C, followed by incubation with DBP (250 μM) for 30 min.

Interestingly, without H_2_O_2_ treatment, HRP still showed cell surface biotinylation, unlike APEX2 (Figure 2A,B). In the imaging experiments, Anti-Myc and Anti-V5 confirmed the cell surface localization of HRP-TM and APEX-TM. Without exogenous H_2_O_2_, cell surface biotinylation was detected in the HRP-TM-expressing cells. We observed that HRP exhibited higher labeling efficiency than APEX2. This can be explained by HRP having a fourfold higher *k*_cat_/*K*_M_ value than APEX2,^32^ and HRP, but not APEX2, has glycosylation^33^ contributing to effective labeling on the plasma membrane. Our findings suggest that HRP-TM could label proximal cells utilizing endogenously generated H_2_O_2_.

In this experiment, we also prepared TurboID-TM-expressed cells for a comparative experiment. It is noteworthy that TurboID, an engineered biotin ligase, has been applied to study astrocyte–neuron interactions at tripartite peri-synapses in the mouse brain. However, TurboID catalyzes the labeling reaction from biotin and ATP and it has been pointed out that the extracellular ATP concentration is 1000-fold lower than the estimated *K*_M_ value of TurboID,^34^ and it is expected that the labeling intensity of TurboID at the cell surface can be highly dependent on the extracellular concentration of ATP, which requires experimental validation. As expected, it was shown that cell surface TurboID had significantly lower labeling efficiency than HRP and APEX2, despite the addition of ATP (Figure S2) possibly due to the active hydrolase activity for ATP at the cell surface.^35^ Since excess treatment of ATP can cause innate immune response by the purinergic receptor,^36^ we excluded the utilization TurboID in our cell–cell labeling study and we focused utilization of HRP for cell–cell interaction labeling.

To confirm that HRP-TM can be labeled using endogenous H_2_O_2_, we generated 293 Flp-In T-REx-293 cell lines that stably express HRP-TM on the cell surface and utilized it to measure the amount of endogenously generated extracellular H_2_O_2_. The amount of H_2_O_2_ generated was quantified using the Peroxide Assay Kit (Figure S4), which can measure the H_2_O_2_ concentration in the liquid sample based on the reaction between iron and H_2_O_2_, which produces ferric ions that react with xylenol orange (XO) to form a colored (purple) complex. The measured H_2_O_2_ concentration in T-REx-293 was about 0.15 μM. In this experiment, we used 12-well plates (surface area: 3.6 cm^2^) and added 1 mL of DPBS, resulting in a height of approximately 2.78 mm. Considering that the cell height is around 10 μm, if we restrict the reaction space to the cell surface membrane, the local concentration of H_2_O_2_ would be approximately 40 μM (∼0.15 μM × 278). We believe that this concentration is sufficient for HRP to function effectively, despite HRP having a *K*_M_ value in the mM range. These measurements confirm the presence of physiologically relevant concentrations of H_2_O_2_ in these cell lines, further supporting the applicability of our labeling platform.

This concentration is sufficient for HRP to function effectively as a substrate. Additionally, we observed a reduction in labeling when H_2_O_2_, the substrate of HRP, was removed by using catalase, indicating the specificity of the labeling (Figure 2C). Similarly, even when exogenous H_2_O_2_ was added, we confirmed that the labeling was reduced due to catalase. To confirm that the endogenously generated H_2_O_2_ was produced by NOX in HEK293,^28–30^ we used the NOX inhibitor ML171 ^37^ (Figure 2D). Upon treatment with ML171, a decrease in HRP labeling was observed. This suggests that endogenous H_2_O_2_ production is indeed mediated by NOX.

Wild-type (WT) cells were stained with the green CMFDA dye in advance and cocultured with HRP-TM-expressing cells for 24 h. Then, the cells were incubated and stained for confocal fluorescence microscopy imaging (Figure 3A). When excessive H_2_O_2_ was treated to cells, diffusive labeling was observed (Figure 3B). The HRP-TM protein passes through the ER and Golgi complexes during its secretory pathway to the cell membrane. This explains the visibility of both the ER and cell membrane patterns. However, in our experiments focused on cell–cell interactions, membrane-localized HRP-TM plays a crucial role. Notably, we observed that without the addition of exogenous H_2_O_2_, neighboring cells to HRP-TM expressing cells were labeled (Figure 3C).

**Figure 3:**
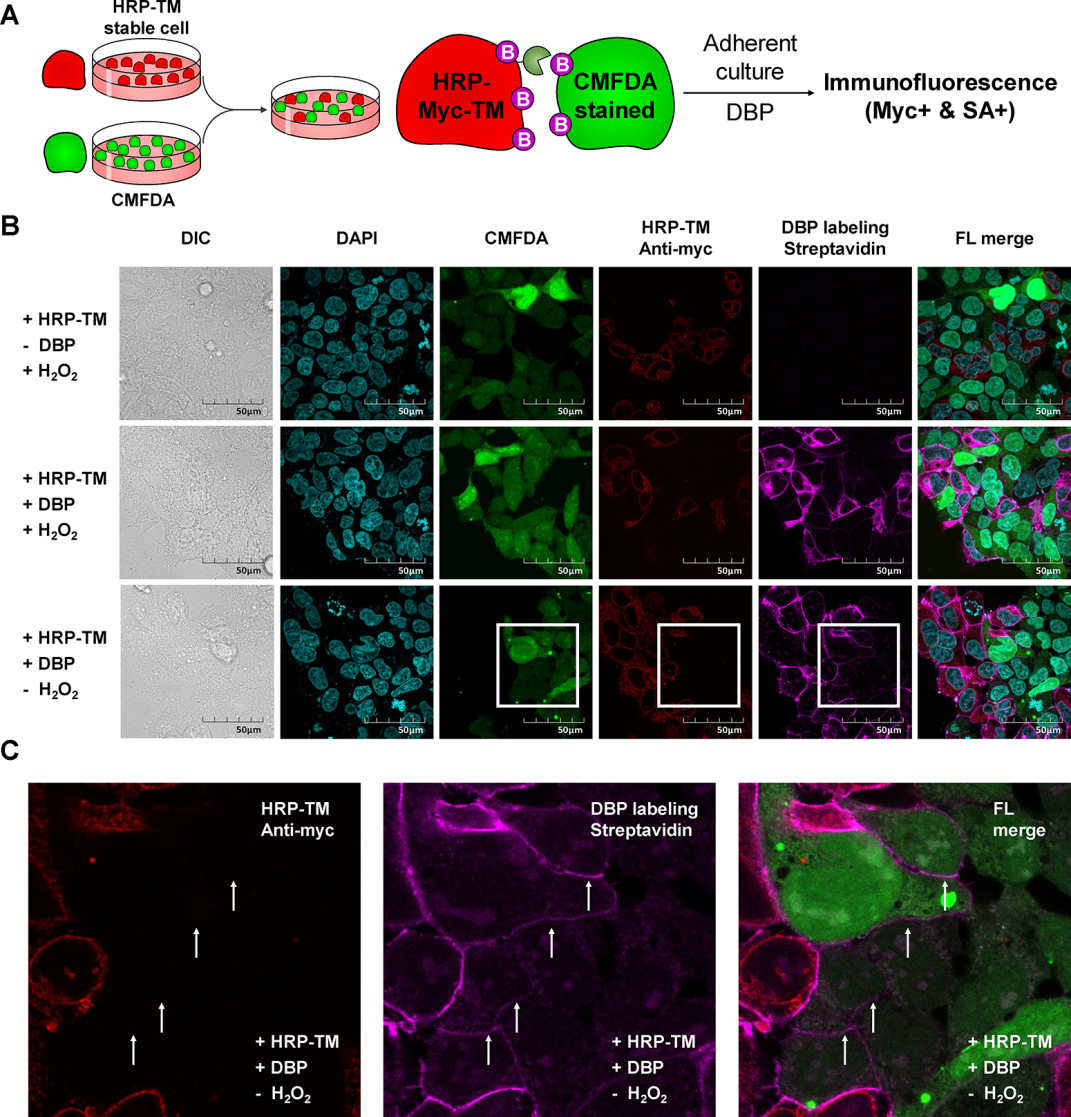
Contact-dependent biotinylation via HRP-TM. (A) HRP-TM stably expressing T-REx-293 cells were cocultured with WT T-Rex-293 cells stained with 1 μM green CMFDA dye, and 250 μM DBP was added for HRP-mediated biotinylation, followed by the addition of 1 mM H_2_O_2_ for 1 min at RT. Cells were stained with DAPI, mouse anti-Myc, antimouse-AF568, and streptavidin-AF647 for fluorescence microscopy. (B) Confocal fluorescence microscopy images of HRP-TM-mediated biotinylation of contacting cells. (C) Zoom-in regions in (B).

We confirmed the generation of endogenous H_2_O_2_ in HEK293T cells and extended this observation to other cell lines (Figures S4A and S5A). Specifically, we established a stable expression of HRP-TM in the malignant glioma cell line U-87MG using a lentiviral system. Similar to the experiments performed in T-REx-293 cells, we quantified the concentration of endogenous H_2_O_2_ using the Peroxide Assay Kit (Figure S4B), finding it to be approximately 0.075 μM. Similar to the T-REx-293 cell calculation, when estimating the local concentration of H_2_O_2_ at the cell surface membrane, it can be gauged to be around 20 μM. Additionally, to confirm endogenous H_2_O_2_ generation in Jurkat T lymphocyte cells, we used a recombinant Wheat Germ Agglutinin (WGA)-HRP (WGA-HRP), which specifically binds to GlcNAc, sialic acid (Figure S5B). This allowed us to verify the labeling mediated by endogenous H_2_O_2_.

Next, to test whether HRP-TM can successfully label interacting cells specifically, we employed two pairs of epitope tag-nanobody pairs to induce intercellular interactions: HAFrankenbody (HAFB, 26.6 kDa)-3xHA tag (YPYDVPDYA)^34^ and ALFAnanobody (NbALFA, 14.5 kDa) -ALFA tag (SRLEEELRRRLTE).^38^ HAFB and NbALFA are known to bind their target tags strongly with high binding affinity of *K*_D_ = 14.7 ± 7.4 nM and *K*_D_ = ∼26 pM, respectively.^38,39^ HAFB (cell A) and HA-tag (cell a) or NbALFA (cell B) and ALFA-tag (cell b) will result in two cells in proximity, thereby biotinylation across the cell–cell interface (Figure 4A).

**Figure 4:**
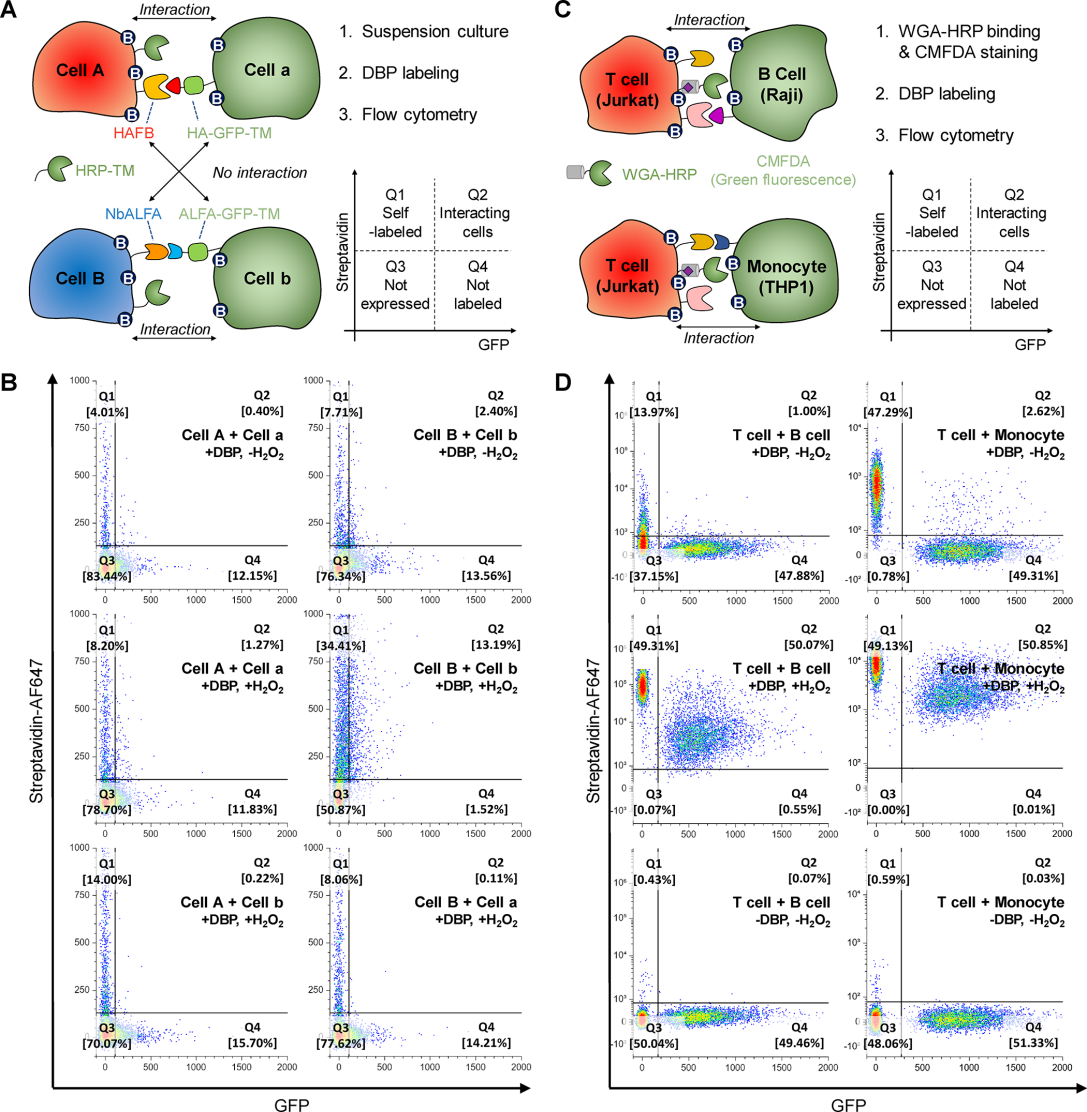
Cell–cell interaction-dependent biotinylation via HRP. (A) Experimental scheme of interaction-dependent biotinylation mediated by HRP-TM. Cell A and cell a were mixed either with cell B or cell b, followed by the sequential addition of DBP. Cells were then stained with streptavidin-AF647 and analyzed by flow cytometry. (B) Flow cytometric analysis of HAFB (αHA)-HA tag and NbALFA (αALFA)-ALFA tag interaction-dependent biotinylation via HRP-TM. (C) Experimental scheme of interaction-dependent biotinylation mediated by wheat germ agglutinin (WGA)-HRP. T cell (Jurkat) was mixed either with B cell (Raji) or monocyte (THP-1). T cells were pretreated with 2 μg/mL of WGA-HRP, and B cells and monocytes were pretreated with 1 μM of CMFDA. Cells were then stained with streptavidin-AF647 and analyzed by flow cytometry. (D) Flow cytometric analysis of T cell–B cell interaction and T cell–monocyte interaction via WGA-HRP.

To assess cell–cell interaction-dependent biotinylation, four groups of cells were prepared. Cell A and cell B were transfected with HRP-TM, along with HAFB-TM and NbALFA-TM, respectively. Cell a and cell b were transfected with 3xHA-GFP-TM or ALFA-GFP-TM, respectively. Cell A and cell B were then either mixed with cell a or cell b, followed by DBP for biotinylation, with or without exogenous H_2_O_2_. The cells were subsequently stained and analyzed via flow cytometry. In this experiment, we also tested the combination of cell A and cell b, or cell B and cell a, which were expected to have no interaction. After single-cell gating (Figure S6A), Q1 (streptavidin positive and GFP negative) represents self-labeled cells by HRP-TM, Q2 (streptavidin positive and GFP positive) represents interacting proximal cells, Q3 (streptavidin negative and GFP negative) represents cells that were not expressed, and Q4 (streptavidin negative and GFP positive) represents HAFB- or NbALFA-expressed cells but not biotin labeled in a flow cytometry graph (Figure 4A). In addition, the labeling efficiency was calculated by dividing the fraction of both GFP and streptavidin positive cells by the total number of GFP-positive cells (Figure S6B).

Under H_2_O_2_ treatment conditions (middle, Figure 4B), both cell A and cell B successfully labeled their interacting proximal cells (Q2). The labeling of proximal cells in the NbALFA–ALFA pair (cell B, cell b, 89.67%) was more efficient than in the HAFB-HA pair (cell A, cell a, 9.69%). In mismatched pairs (cell A–cell b, cell B–cell a), almost no biotinylation (1.38%, 0.77%) was observed even under H_2_O_2_ treatment (bottom, Figure 4B), indicating the interaction specificity between two ligand–receptor pairs. Interestingly, matched cells (cell A–cell a, cell B–cell b) were biotinylated (3.19%, 15.04%) even without exogenous H_2_O_2_ (top, Figure 4B). Consistent with other data, HRP-TM labeled the proximal cells in an interaction-specific manner by utilizing endogenously generated H_2_O_2_.

In addition to artificially induce interactions using epitope tags, we aimed to verify spontaneous interactions between immune cells. To achieve this, we coated the surface of T cells with HRP and sought to detect their interactions with B cells and monocytes through biotinylation. We attached WGA-HRP to the glycans on the surface of T cells and incubated B cells and monocytes with GFP fluorescent CMFDA dye. As in the previous experiments, T cells were mixed with either B cells or monocytes, followed by DBP for biotinylation, with or without exogenous H_2_O_2_. The cells were subsequently stained and analyzed by flow cytometry. After single-cell gating (Figure S6B), Q1 (streptavidin positive and GFP negative) represents self-labeled cells by WGA-HRP, Q2 (streptavidin positive and GFP positive) represents interacting proximal cells, Q3 (streptavidin negative and GFP negative) represents cells where DBP labeling was insufficient in T cells, and Q4 (streptavidin negative and GFP positive) represents B cells and monocytes that were not biotin labeled in the flow cytometry graph (Figure 4C). Additionally, the labeling efficiency was calculated by dividing the fraction of both GFP- and streptavidin-positive cells by the total number of GFP-positive cells (Figure S7B).

Under H_2_O_2_ treatment (middle, Figure 4D), T cells successfully labeled their interacting proximal cells (Q2). Unlike the transfection experiments, where interactions were limited, immune cells were able to label interacting cells in a many-to-many manner (Q2/Q2+Q4:98.91% in the T cell–B cell labeling experiment, 99.98% in the T cell-monocyte labeling experiment). Even without exogenous H_2_O_2_, interacting cells (T cell–B cell and T cell-monocyte) were biotinylated (Q2/Q2+Q4:2.05%, 5.05%) (top, Figure 4D) and this interaction was reproducibly shown in the replicate experiments (Figure S8). Although they did not produce as much endogenous H_2_O_2_ as T-REx-293or U-87MG cells under normal conditions, it was still possible to identify interacting cells. This WGA-HRP system has the potential to be widely applied and is expected to be particularly useful in *in vitro* reaction settings.

## Discussion

In this study, we showed that HRP, not APEX2, can facilitate endogenously generated H_2_O_2_ on the cell surface and label proximal cells. Our findings imply that HRP-mediated biotinylation can be used for *in vivo* applications on the cell surface to detect cell–cell networks without toxic H_2_O_2_ treatment. Several studies have used HRP for cell-surface proteome profiling. Li et al.^40^ developed an HRP-based technique for cell-surface proteome profiling in the brain tissue of *Drosophila*. Shuster et al.^34^ applied HRP-mediated cell-surface proteomics to the mouse brain. Since HRP showed no toxicity issue in their expression in live animal model in these studies,^31,36^ we expect that our cell–cell interaction labeling technique can be employed in these HRP-expressed animal models. We believe that HRP-mediated proximal cell labeling has advantages in overcoming the limitations of other approaches, such as utilization of high molecular weight of probe (LIPSTIC, EXCELL, PUP-IT, FucoID),^9–12^ light irradiation (μMap, PhoTag, PhoXCELL, Ru-^1^O_2_-hydrazide),^15–18^ low concentration of extracellular ATP (TurboID),^41^ and exogenous treatment of H_2_O_2_ (APEX2).

While utilizing endogenously generated H_2_O_2_ for initiating HRP-mediated labeling offers advantages, there are some caveats associated with this approach. First, the production of H_2_O_2_ can vary across different cell types. Second, even within the same cell type, the levels of H_2_O_2_ production may fluctuate under different cellular states. These factors can influence the effectiveness of capturing cell–cell interactions. For example, variations in the quantity of H_2_O_2_ produced by cells before and after drug treatment may lead to changes in the labeling of the interacting cells. Such fluctuations may not accurately represent the cell interactions themselves but rather arise from shifts in the H_2_O_2_ production levels. Third, labeling efficiency can vary depending on experimental conditions. Labeling is most effectively observed under adherent coculture conditions. In single culture conditions, labeling can vary with cell confluency, potentially resulting in intracellular labeling rather than labeling of contacting cells. In suspension coculture conditions, labeling efficiency may also vary depending on the probability of cell–cell contact. Lastly, we recognize that differences in HRP-TM expression levels in our transfection-based experiments may have influenced the efficiency of our results. To address this issue, we either created stable cell lines for our experiments or introduced a method utilizing recombinant WGA-HRP. Without the need to introduce a new gene into the cells, WGA-HRP can be applied to bind the sialic acid present on the surface of most mammalian cells.^42^ Leveraging this versatility, we propose the potential application of this method in studying cell–cell interactions in primary or patient-derived cells.

Recently, an integrated approach utilizing proximity labeling and omics data has been reported. Oslund et al.^16^ combined PhoTag with multiomics single-cell sequencing and discovered a specific T cell subtype in human peripheral blood mononuclear cell (PBMC) that interacted more with Raji B cells. Zhang et al.^43^ applied QMID to the mouse spleen and characterized gene expression profiles of CD4+ or CD8+ proximal cells by single-cell RNA sequencing (scRNA-Seq). However, these methods are limited to *ex vivo* models, thus prompting increased interest in developing a tool suitable for *in vivo* models. In this context, Hamachi et al. proposed a new bacterial tyrosinase-based PL method, emphasizing its efficient application *in vivo.* ^44^ In this Letter, we propose the possibility that HRP-TM could paint proximal cells in *in vivo* models by utilizing endogenously generated H_2_O_2_. Exploring the interaction between viruses and cells is also an interesting theme, as viruses rely on receptor proteins to invade host cells.^45^ By integrating the outcomes of HRP-mediated cell labeling with additional spatial omics data, such as single-cell RNA sequencing (scRNA-seq), we anticipate a comprehensive elucidation of the intricate cell–cell network implicated in disease progression. This approach promises to provide valuable insights into the dynamic cellular landscape underlying various pathological conditions.

## Supplementary Material



## Notes

Materials and methods, construct information, and additional experimental figures including immunofluorescence microscopy images and Western blot (PDF)

## #

Department of Chemistry, Princeton University, Princeton, New Jersey 08544, USA

## Abbreviations

HRP: horseradish peroxidase
TM: transmembrane domain
BP: biotin-phenol
DBP: desthiobiotin-phenol
CMFDA: 5-chloromethylfluorescein diacetate
GFP: green fluorescent protein
WGA: wheat germ agglutinin.
