# Supplementary material for: Painting Cell–Cell Interactions by Horseradish Peroxidase and Endogenously Generated Hydrogen Peroxide
Source: ACS Chem Biol. 2024 Dec 18;20(1):86–93. doi: 10.1021/acschembio.4c00419 (PMC13459412; doi:10.1021/acschembio.4c00419)
Supplement: Supplementary file 1 [file cb4c00419_si_001.pdf]

Supporting Information

Painting cell-cell interactions by horseradish peroxidase and endogenously generated hydrogen peroxide

Youngjoon Cho[1,†], Inyoung Jeong[1,†,#], Kwang-eun Kim[1,2*], and Hyun-Woo Rhee[1,3*]

[1]Department of Chemistry, Seoul National University, Seoul 08826, Korea

[2]Department of Convergence Medicine, Yonsei University Wonju College of Medicine, Wonju 26426, Korea

[3]School of Biological Sciences, Seoul National University, Seoul 08826, Korea

[†]These authors equally contributed to this work.

<u>Table of contents</u>

Material and Methods S2

Table S1: Construct information........ S6

Figure S1: Comparison of peroxidase labeling activity at the cell surface following H_2O_2 treatment S8

Figure S2: Activity of TurboID at cell surface with or without ATP........ S9

Figure S3: Negative control of contact-dependent biotinylation using HRP-TM........ S10

Figure S4: Measurement of endogenous H_2O_2 levels........ S11

Figure S5: Labeling of various cell types via HRP S12

Figure S6: Gating strategy used in flow cytometry analysis........ S13

Figure S7: Calculation of labeling efficiency in interacting cells S14

Material and Methods

Plasmid cloning

Genes were amplified through PCR (Bioneer), digested with restriction enzymes (NEB, Enzynomics), and ligated into plasmid vectors with T4 ligase (Enzynomics). The SP-HRP-Myc-TM_pCDNA5, SP-TurboID-Myc-TM_pDisplay, SP-HRP-Myc-TM_pLX304 genes were subcloned from SP-HA-HRP-Myc-TM_pDisplay, and V5-APEX2-TM_pCDNA5 was subcloned. SP-3xHA-EGFP-TM, SP-ALFA-EGFP-TM, SP-HAFrankenbody(α-HA)-V5-Halotag-TM, and SP-NbALFA(α-ALFA)-V5-Halotag-TM in pDisplay vectors were subcloned from SP-HA-HRP-Myc-TM_pDisplay. The HAFraneknbody gene was amplified from pET23b-15F11-HA-mEGFP-6xHis was a gift from Tim Stasevich (Addgene plasmid # 129593 ; http://n2t.net/addgene:129593 ; RRID:Addgene_129593). The NbALFA gene was amplified from EGFP-NbLAFA_pET51b(+), which was a gift from Kai Johnsson (Addgene plasmid # 136626 ; http://n2t.net/addgene:136626 ; RRID:Addgene_136626).

General cell culture and transfection

HEK293T cell line was purchased from ATCC (Manassas, VA, USA), and Flp-In T-REx-293 cell line was purchased from Thermo Fisher Scientific. All cells were cultured in DMEM with 4.5 g/L glucose, L-glutamine, and sodium pyruvate (Corning, 10-013-CVC) supplemented with 10% FBS (Atlas EquaFETAL, US Origin Bovine Serum, EF-0500-A) and 100 U/mL penicillin-Streptomycin (Thermo Fisher Scientific, 15140-122). Cells were grown at 37 °C with 5 % CO_2. For confocal microscopy imaging, cells were grown on poly-L-Lysine coated 18 mm glass coverslips in 12-well plates (NEST). For Western blotting, cells were grown in 12-well and 6-well plates. For flow cytometry, cells were cultured in 6-well plates (NEST) or T25-cell culture flask (NEST). Cells were transfected at 60-80 % confluence using polyethylenimine (PEI) and 1,000 ng plasmid DNA in 100 μL serum-free media per 3×10^5 cells for 4-8 h, then the media was replaced with fresh DMEM.

Stable cell line generation (Flp-In™ system)

For a generation of cell lines that stably express Igκ leader signal peptide (SP)-HRP-Myc-TM, Flp-In T-REx-293 cells were grown in a 6-well plate. Cells were transfected with 200 ng of SP-HRP-Myc-TM subcloned into pCDNA5 vector, and 1800 ng of pOG44 vector and PEI for 4 h, after which the media was replaced with fresh culture media. After 24 h, cells were trypsinized and cultured in a 100 pi cell-culture dish (SPL, 20100) for another 24 h. Then the media was replaced with fresh DMEM containing 500 ug/mL Geneticin (G418) every three days. After 3 weeks, single colonies were selected and transferred into a 24-well plate and cultured for a larger scale. Generated stable cell lines were confirmed through Western blotting and immunofluorescence, and cell stocks were stored at -80 °C. Expression of SP-HRP-Myc-TM proteins was induced by treating 5 ng/mL doxycycline (Sigma Aldrich) for over 16 h.

Stable cell line generation (Lenti viral infection)

For a generation of cell lines that stably express Igκ leader signal peptide (SP)-HRP-Myc-TM, HEK293T cells were grown in a 6-well plate. Cells were transfected with 1000 ng of SP-HRP-Myc-TM subcloned into pLX304

vector, 750 ng of psPAX2 (Addgene #12260), 250 ng of pMD2G (Addgene #12259) using PEI for 4 h. After 4 h, the media was replaced with fresh culture media. After 24 h, viral supernatant was harvested and passed through a PES 0.45 μm filter (JET-BioFil, FPE-404-013). U-87MG cells were grown in a 6-well plate and replaced with filtered viral media with hexadimethrine bromide (5 μg/mL) in 60~80% cell confluence. After 24 h, the media was replaced with fresh culture media. After 24 h, cells were trypsinized and cultured in a 100pi cell-culture dish (SPL,20100) for another 24 h. Then the media was replaced with fresh culture media containing 10 μg/mL blasticidin. After 3 weeks, single colonies were selected and transferred into a 24-well plate and cultured for a larger scale. Generated stable cell lines were confirmed through western blotting and immunofluorescence.

Catalase treatment

HRP-TM stably expressing T-REx-293 cells were grown in 6 well plates. Expression of SP-HRP-Myc-TM proteins was induced by treating 5 ng/mL doxycycline (Sigma Aldrich). After 24 h, a culture medium was replaced with 10 μg/mL catalase (Sigma, C1345) and 250 μM desthiobiotin-phenol containing media and incubated for 30 min at 37°C, followed by the addition of 1 mM H_2O_2 for 1 min at RT. The HRP-TM labeling was terminated by replacing the media with quencher solution containing 5 mM Trolox (Sigma Aldrich), 10 mM Sodium Azide (TCI), 10 mM Sodium Ascorbate (Sigma Aldrich). Cells were washed one more time with quencher solution and two more times with DPBS. Cells were lysed with RIPA buffer solution (Pierce, 89901) supplemented with protease inhibitor cocktail (HALT, 78438) at 4 °C for 10 min, followed by centrifugation at 15,000 g for 10 min at 4 °C to remove cell pellet and harvest clear supernatant.

NOX inhibitor treatment

HRP-TM stably expressing T-REx-293 cells were grown in 6 well plates and expression of SP-HRP-Myc-TM proteins was induced by treating 5 ng/mL doxycycline (Sigma Aldrich) media. After 24 h, cells were incubated ML171(Sigma, 492002) containing media for 1 h at 37°C. For HRP-TM biotinylation, cells were replaced with ML171 and 250 μM desthiobiotin-phenol containing media and incubated for 30 min at 37°C, followed by the addition of 1 mM H_2O_2 for 1 min at RT. The HRP-TM labeling was terminated by replacing the media with a quencher solution. Cells were washed one more time with quencher solution and two more times with DPBS. Cells were lysed with RIPA buffer solution containing protease inhibitor cocktail at 4°C for 10 min, followed by centrifugation at 15,000 g for 10 min at 4 °C to remove cell pellet and harvest clear supernatant.

***In vitro* biotinylation and cell lysate preparation**

Biotin was prepared from a 5 mM Biotin (Sigma, B4639) dissolved in PBS. TurboID-expressing cells were cultured at 37 °C for 30 min in a culture medium supplemented with 50 uM biotin, and 1 mM ATP (Sigma Aldrich) and 5 mM $MgCl_2$ (Sigma Aldrich) were added for TurboID-mediated biotinylation in the extracellular matrix. Cells were then washed three times with Dulbecco's phosphate-buffered saline (DPBS), pH 7.4. For HRP and APEX2-mediated biotinylation, cells were incubated with a culture medium containing 250 μM desthiobiotin-phenol (DBP) at 37 °C for 30 min. The peroxidase reactions were initiated by adding 1 mM H_2O_2 for 1 min at RT and terminated

by replacing the media with Quencher solution. Cells were washed one more time with Quencher solution and two more times with DPBS. Cells were lysed with RIPA buffer solution supplemented with Protease Inhibitor Cocktail at 4 °C for 10 min, followed by centrifugation at 15,000 g for 10 min at 4 °C to remove cell pellet and harvest clear supernatant.

Proximal cell surface biotinylation via HRP-TM

HEK293T cells were cultured in a 6-well plate for 24 h. HEK293T cells were stained with 1 uM CellTracker™ Green CMFDA Dye (Invitrogen, C7025) in a culture medium for 30 min, followed by DPBS wash three times. After 24 h, HEK293T cells were trypsinized and mixed with HRP-TM stably expressing T-REx-293 cells in a ratio of 2:1 in cell numbers and co-cultured in the poly-L-Lysine coated 18 mm glass coverslips in 12-well plate, with a culture medium containing 5 ng/mL of doxycycline, to induce HRP-TM expression. After 18 h, a culture medium was replaced with fresh DMEM supplemented with 250 μM DBP for 30 min at 37°C, followed by the addition of 1 mM H_2O_2 for 1 min at RT. Cells were rinsed three times with DPBS and fixed with 4 % paraformaldehyde (PFA) (Chembio, CBPF-9004-1000) at RT for 10 min, followed by DPBS wash two times.

Cell surface biotinylation via WGA-HRP

2×10^6 cells/mL of Jurkat cells in 100 pi dish were incubated with 2 μg/ml WGA-HRP at 37°C for 30 min. Cells were centrifuged in 300 g for 5 min and washed with DPBS. Cells were divided to 6 wells cell culture plate and incubated with 250 μM DBP for 30min at 37°C, followed by centrifugation at 300g for 5min. Cells were washed with DPBS and lysed with RIPA buffer solution supplemented with Protease Inhibitor Cocktail.

Cell-cell interaction-dependent biotinylation *in vitro* and flow cytometry

For cell-cell interaction-dependent biotinylation, 1.5×10^6 of HEK293T cells were cultured in T25-cell culture flasks for 24 h. HEK293T cells were co-transfected with SP-HRP-Myc-TM and the following plasmids respectively to generate Cell A, Cell B, Cell a, and Cell b, by the method described above: SP-HAFB-V5-Halotag-TM (Cell A), SP-NbALFA-V5-Halotag-TM (Cell B), SP-3xHA-EGFP-TM (Cell a), SP-ALFA-EGFP-TM (Cell b). After 18 h, cells were trypsinized and centrifuged in 196 g for 3 min to get cell pellets. Cells were reconstituted with 1.5 mL culture media. Either 225 μL of Cell A or Cell B and another 225 μL of Cell a or Cell b were mixed in new e-tubes and co-incubated at RT for 2 h in a rotator. DBP was added at a final concentration of 50 μM at RT for 30 min in a rotator followed by adding 50 μL of either DPBS or H_2O_2 at a final concentration of 1mM at RT for 1 min for HRP-mediated biotinylation. Then the reaction was terminated by adding the Quencher solution. Cells were rinsed with DPBS two times immediately using centrifugation and rinsed one more time for 2 h at RT in a rotator to remove unreacted free DBP. Then, cells were stained with Streptavidin-Alexa Fluor 647 (Invitrogen, 1:1,000) in DPBS at RT for 20 min and analyzed by FACS Canto II (BD Biosciences).

For cell-cell interaction-dependent biotinylation, 2×10^6 of T cell (Jurkat) were incubated with 2ug/ml of WGA-HRP and 1×10^6 of B cell (Raji), monocyte (THP-1) cell were stained with 1 μM of CMFDA dye for 30 min, followed by centrifugation at 300 g for 5 min. Cells were washed with DPBS and diluted to 3×10^6 cells/mL. 225

μL of T cell and another 225 μL of B cell or monocyte were mixed in new e-tubes and co-incubated with DBP containing tube at RT for 1 h in a rotator, followed by adding 50 μL of either DPBS or H_2O_2 at a final concentration of 1 mM at RT for 1 min for HRP-mediated biotinylation. Then the reaction was terminated by adding the quencher solution. Cells were rinsed with DPBS two times immediately using centrifugation. Then, cells were stained with Streptavidin-Alexa Fluor 647 (Invitrogen, 1:1,000) in DPBS at RT for 20 min and analyzed by FACS Canto II (BD Biosciences).

Western Blotting

Protein concentration in lysates was measured by BCA assay (Pierce, 23225). Equal amounts of proteins were prepared and denatured by adding 5x SDS-PAGE loading-Buffer (Biosesang) and boiling at 95 °C for 5 min. Denatured proteins were loaded and separated by 8-10 % SDS-PAGE gels, which were transferred to the Nitrocellulose membrane (Pall Incorporation) after separation. Membranes were stained with Ponceau S for 3 min and washed three times with TBST (25 mM Tris, 150 mM NaCl, 0.1 % Tween 20, pH7.5) for 5 min each. Membranes were blocked with 2 % skim milk at RT for 1 h, followed by primary-antibody incubations, Anti-V5 (Invitrogen, R960-25, 1:10,000), Anti-Myc (Cell Signaling Technology, 2276S, 1:1,000) in 2 % skim milk at RT for 1 h or at 4°C for overnight. After washing three times with TBST for 5 min each, membranes were incubated with secondary-antibodies, anti-mouse-HRP antibodies (CellNest, 1:3,000) in 2 % skim milk and Streptavidin-HRP (Thermo Fisher Scientific, 1:10,000) in TBST was incubated at RT for 1 h and washed three times for 5 min each. Membranes were incubated with ECL for chemiluminescence and detected by IQ800.

Immunofluorescence

Fixed cells were blocked with 2 % bovine serum albumin (BSA) (Bovogen, BSAS0.1) filtered through a 0.2 μm syringe filter, at RT for 1 h. Cells were incubated with primary antibodies, anti-Myc (Cell Signaling Technology, 2276S, 1:1,000) in filtered 2 % BSA at 4 °C overnight. After being rinsed three times with DPBS, cells were incubated with secondary antibodies, anti-mouse-Alexa Fluor 568 antibody (Invitrogen, 1:3,000) in filtered 2 % BSA and Streptavidin-647 (Invitrogen, 1:3,000) at RT for 1 h, followed by a subsequent wash with DPBS for three times. Cells were imaged using FV-3000 confocal microscope (Olympus), with 405, 488, 568, and 647 nm lasers.

Measurement of endogenous H_2O_2 levels

HRP-TM stably expressing T-REx-293 cells and U-87MG cells were seeded into three wells each of a 12-well plate. For the T-REx-293 cells, doxycycline induction was performed to induce HRP-TM expression. Once the cells reached 100% confluency, the media was removed, and the cells were washed twice with Dulbecco's Phosphate-Buffered Saline (DPBS). Subsequently, 1 mL of DPBS was added to each well. Simultaneously, hydrogen peroxide (H_2O_2) solutions were prepared at concentrations of 1, 2, 3, and 4 μM. The cells and H_2O_2 standards were incubated at 37 °C for 30 min. During the incubation period, the working reagent (WR) for the Pierce™ Quantitative Peroxide Assay Kit (ThermoFisher, 23280) was prepared by mixing Reagent A (25 mM ammonium ferrous (II) sulfate in 2.5 M H_2SO_4) and Reagent B (100 mM sorbitol and 125 μM xylenol orange in

water) in a ratio of 1:100. After the 30 min incubation, the H_2O_2 standards and cell samples were mixed with the WR at a ratio of 1:10 and incubated at room temperature for 15 min. The absorbance was measured at 595 nm using a Synergy H1 microplate reader (BioTek). A standard curve was generated from the absorbance values of the 1–4 μM H_2O_2 standards, which was then used to calculate the H_2O_2 concentrations in the cell samples.

Table S1. Construct Information

Name (expected size)	Features	Promoter/Vector	Details	Note
SP-HRP-Myc-TM (398 a.a., 43.9 kDa)	*AflII-HindIII*-Igk SP-*ApaI*-HRP-*SacII*-Myc-PDGFRβ TM-Stop-*NotI-XhoI*	CMV/pCDNA5	**Igk**: METDTLLLWVLLLWVPGSTGD<br>**Myc:** EQKLISEEDL<br>**TM:** VGQDTQEVIVVPHSLPFKVVVI SAILALVVLTIISLIILIMLWQKK PR	Subcloned from the plasmid obtained from addgene (Plasmid# 44441).
SP-V5-APEX2-TM (336 a.a., 36.3 kDa)	*NotI*- Igk SP-V5-APEX2-*NheI*-PDFGRβ TM-Stop-*XhoI*	CMV/pCDNA5	Igk: METDTLLLWVLLLWVPGSTGD<br>V5: GKPIPNPLLGLDST<br>TM: AVGQDTQEVIVVPHSLPFKVVV I SAILALVVLTIISLIILIMLWQKK PR	Mishra et al., *Anal. Chem*. 2022; PMID: 36265183
SP-TurboID-Myc-TM (409 a.a., 44.7 kDa)	*EcoRV*- Igk SP-*ApaI-AflII*-TurboID-AgeI-*SalI*-Myc-PDGFRβ TM-Stop-*NotI-XhoI*	CMV/pDisplay	Igk: METDTLLLWVLLLWVPGSTGD<br>Myc: EQKLISEEDL<br>TM: AVGQDTQEVIVVPHSLPFKVVV I SAILALVVLTIISLIILIMLWQKK PR	
SP-3xHA-EGFP-TM (347 a.a., 39.0 kDa)	*EcoRV*- Igk SP-*ApaI*-3xHA-*MluI*-*AflII*-EGFP-*NheI*-*SalI*-PDGFRβ TM-Stop-*NotI-XhoI*	CMV/pDisplay	Igk: METDTLLLWVLLLWVPGSTGD<br>HA: YPYDVPDYA<br>TM: AVGQDTQEVIVVPHSLPFKVVV I SAILALVVLTIISLIILIMLWQKK PR	Used as ligand protein for HAFrankenbody-TM
SP-HAFB-V5-Halotag-TM (644 a.a., 70.8 kDa)	*EcoRV*- Igk SP-*ApaI-BglII*-NbALFA-*AflII*-V5-Halotag-*NheI*-*SalI*-PDGFRβ TM-Stop-*NotI-XhoI*	CMV/pDisplay	Igk: METDTLLLWVLLLWVPGSTGD<br>V5: GKPIPNPLLGLDST<br>TM: AVGQDTQEVIVVPHSLPFKVVV I SAILALVVLTIISLIILIMLWQKK PR	Used as receptor protein for HA tag. HAFrankenbody sequence was obtained from addgene (Plasmid #129590)
SP-ALFA-EGFP-TM (331 a.a., 37.2 kDa)	*EcoRV*- Igk SP-*ApaI*-ALFA-*AflII*-EGFP-*NheI-SalI*-PDGFRβ TM-Stop-*NotI-XhoI*	CMV/pDisplay	Igk: METDTLLLWVLLLWVPGSTGD<br>ALFA: SRLEEELRRRLTE<br>TM: AVGQDTQEVIVVPHSLPFKVVV I SAILALVVLTIISLIILIMLWQKK PR	Used as ligand protein for NbALFA.
SP-NbALFA-V5-Halotag-TM (529 a.a., 58.6 kDa)	*EcoRV*- Igk SP-*ApaI-BglII*-NbALFA-*AflII*-V5-Halotag-*NheI*-*SalI*-PDGFRβ TM-Stop-*NotI-XhoI*	CMV/pDisplay	Igk: METDTLLLWVLLLWVPGSTGD<br>V5: GKPIPNPLLGLDST<br>TM: AVGQDTQEVIVVPHSLPFKVVV I SAILALVVLTIISLIILIMLWQKK PR	Used as receptor protein for ALFA tag. NbALFA sequence was obtained from addgene (Plasmid #136626)

SP-HRP-Myc-TM (398 a.a., 43.9 kDa)	*BsiW*-Igk SP-*ApaI*-HRP-*SacII*-Myc-PDGFRβ TM-Stop-*AgeI*	CMV/pLX304	**Igk**: METDTLLLWVLLLWVPGSTGD **Myc:** EQKLISEEDL **TM:** VGQDTQEVIVVPHSLPFKVVVISAILALVVLTIISLIILIMLWQKKPR	Subcloned from the plasmid obtained from addgene (Plasmid# 44441).

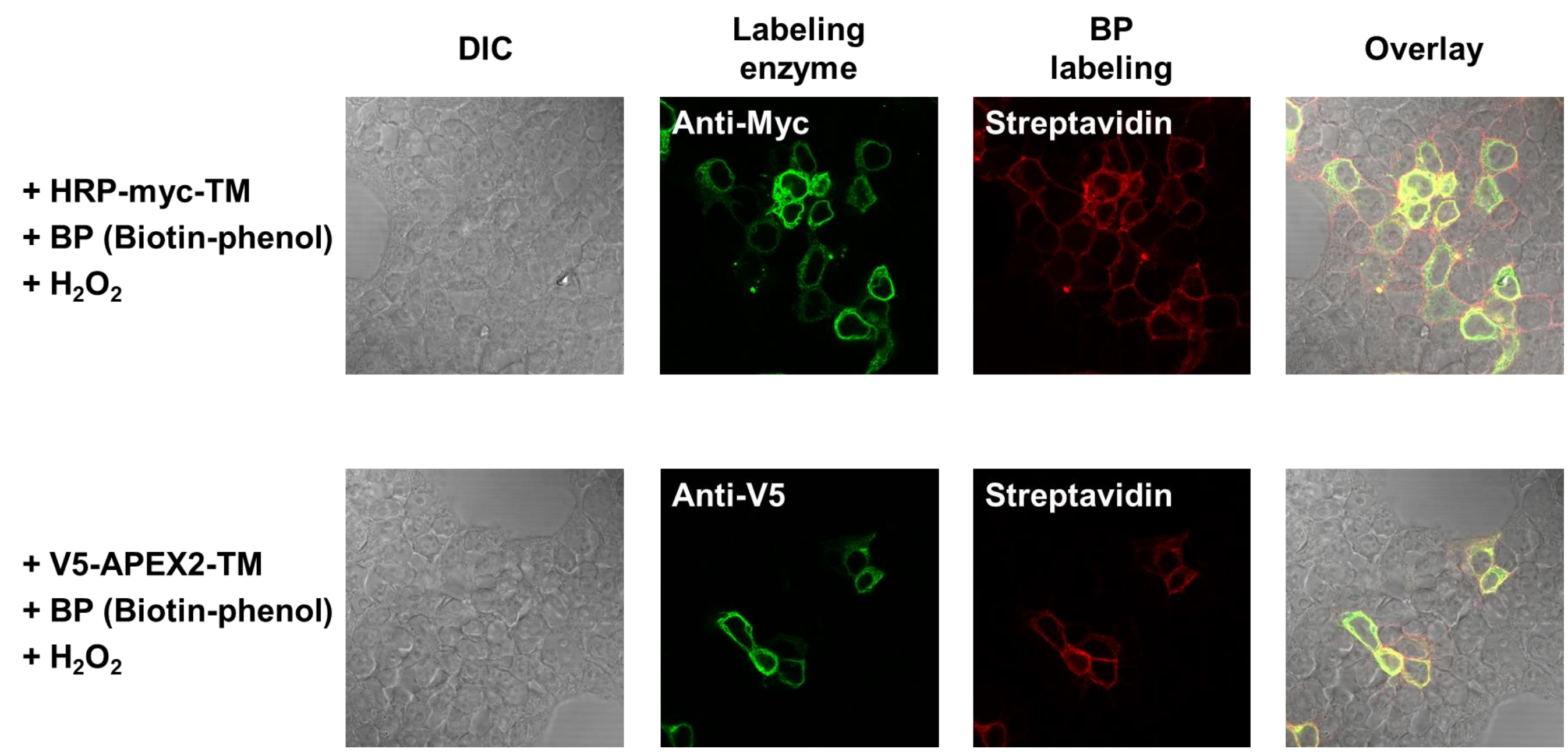

Figure S1. Comparison of peroxidase labeling activity at the cell surface following H_2O_2 treatment

Immunofluorescence imaging HRP-TM (Anti-Myc) or APEX2-TM (Anti-V5), biotinylated proteins (Streptavidin-Alexa) and overlay. HEK293T cells were transfected with HRP-TM, APEX2-TM. Labeling condition: Biotin-phenol 250 µM, 30 min, 37 °C. H_2O_2 1 mM, 1 min, RT

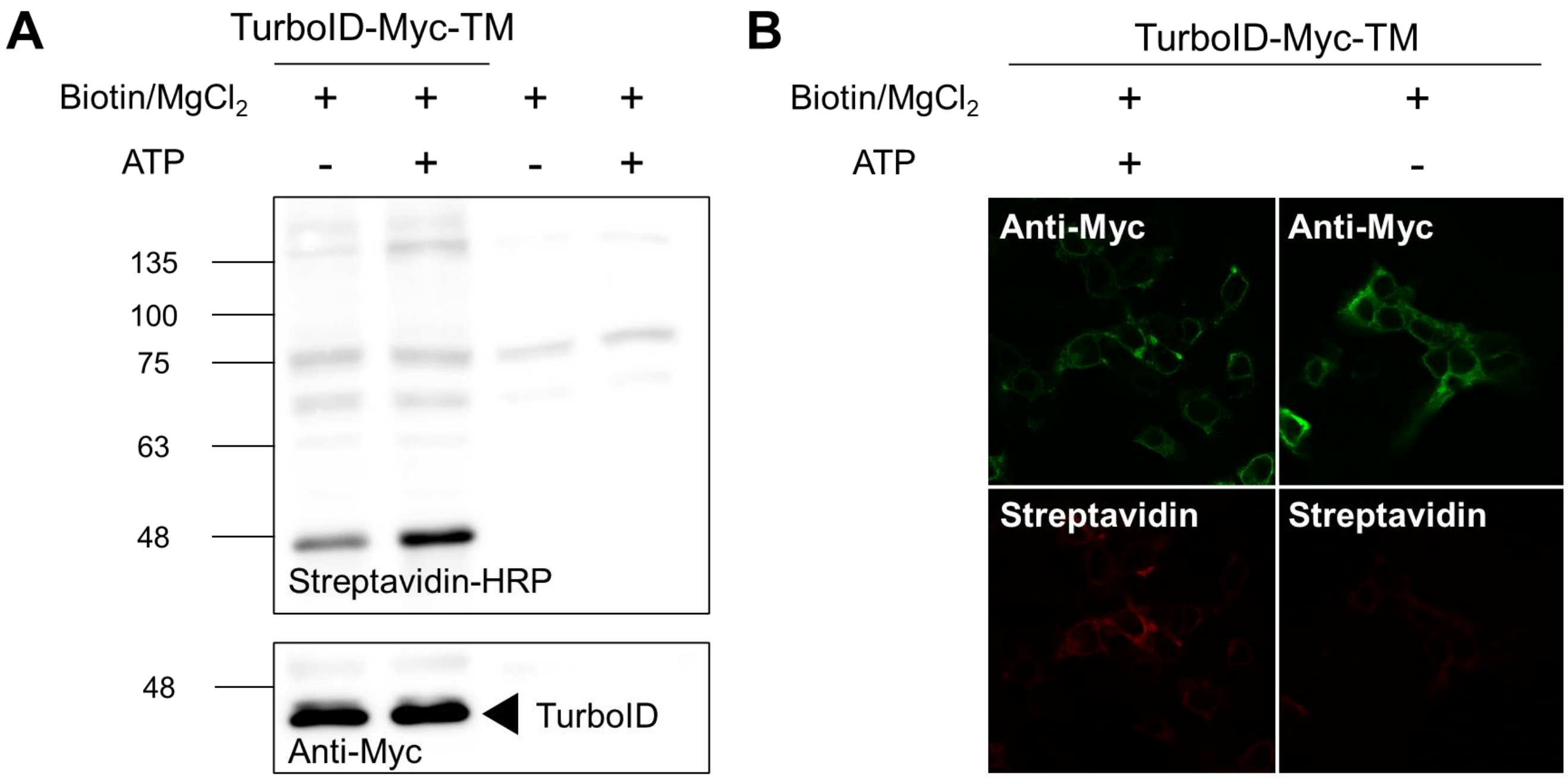

Figure S2. Activity of TurboID at cell surface with or without ATP.
(A) Western blots of biotinylated proteins (Streptavidin-HRP) labeled by TurboID with or without ATP. (B) Immunofluorescence imaging TurboID-TM (Anti-Myc) and biotinylated proteins (Streptavidin-Alexa). HEK293T cells were transfected with TurboID-Myc-TM. Labeling condition: 50 µM biotin, and 1 mM ATP and 5 mM $MgCl_2$.

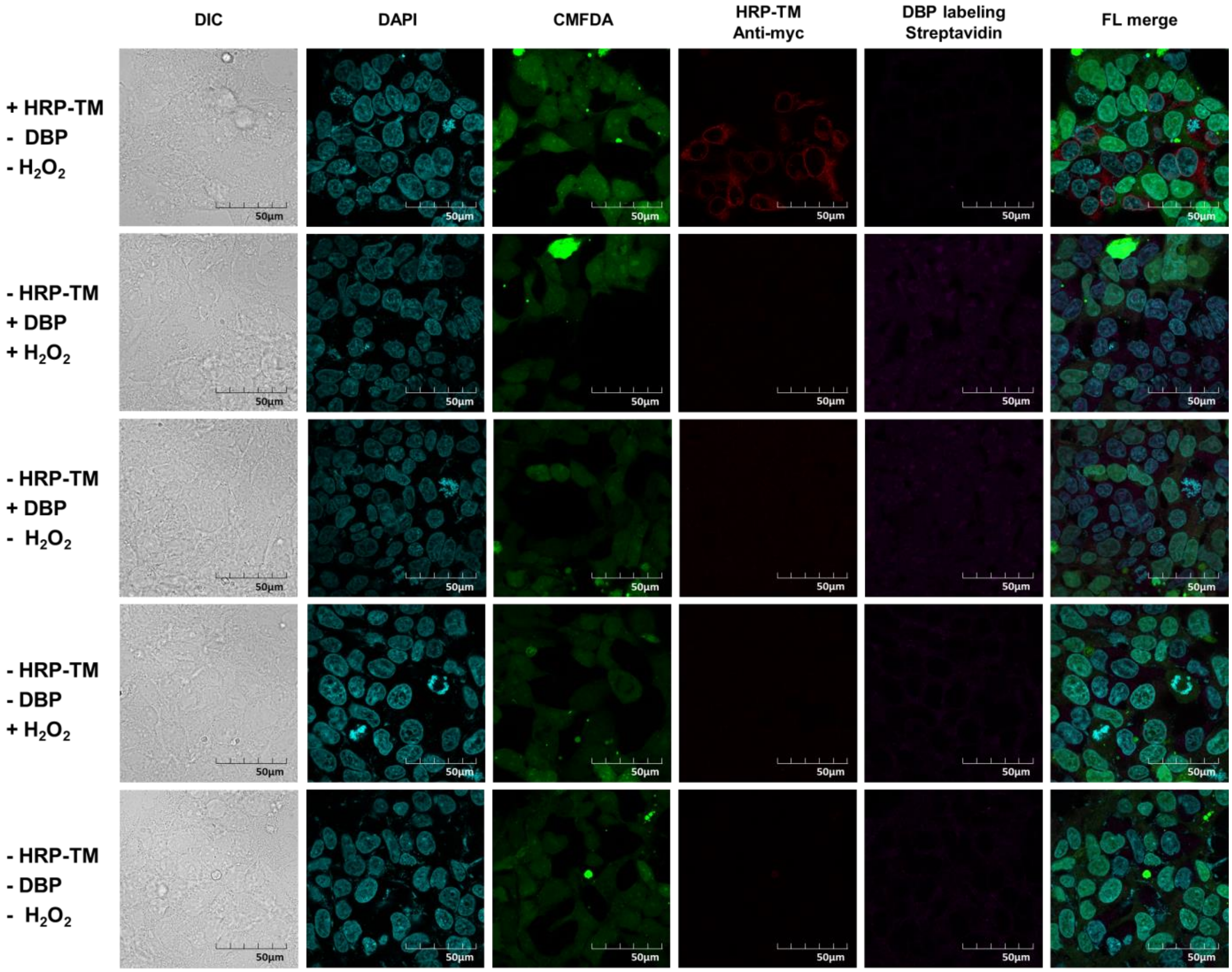

Figure S3. Negative control of contact-dependent biotinylation using HRP-TM

HRP-TM stably expressing T-REx-293 cells were co-cultured with WT T-REx-293 cells stained with green CMFDA. Immunofluorescence imaging HRP-TM (Anti-Myc), biotinylated proteins (Streptavidin-Alexa) and overlay. Labeling condition: DBP 250 µM, 30 min, 37°C. H_2O_2 1 mM, 1 min, RT

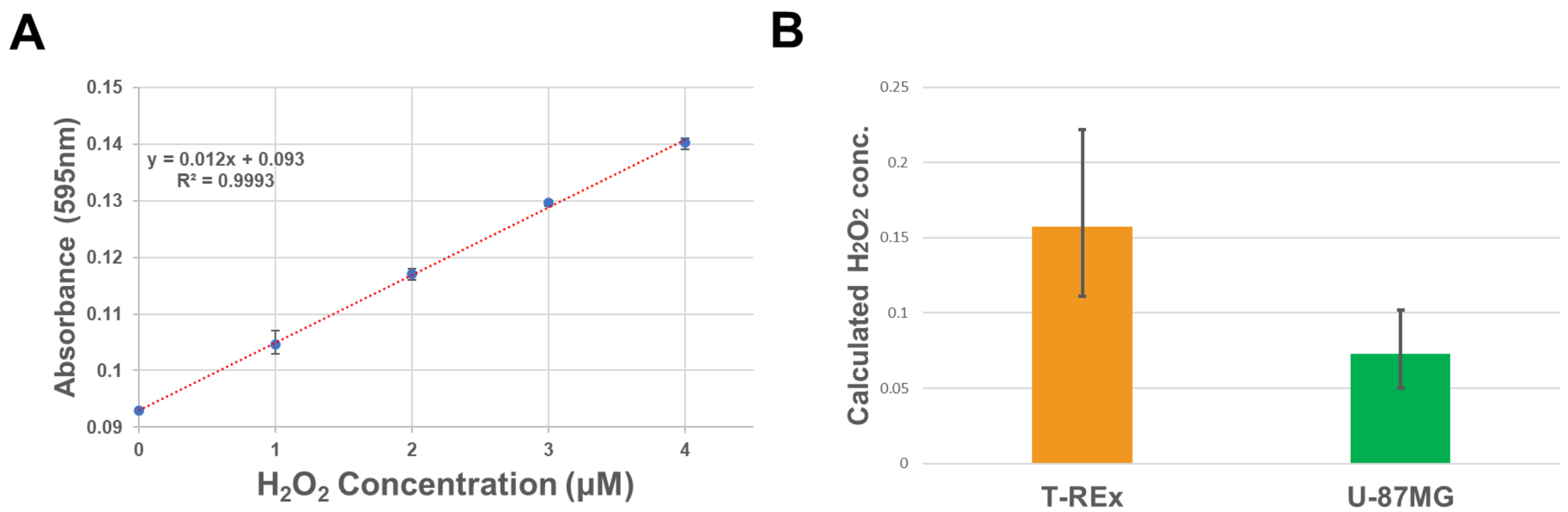

Figure S4. Measurement of endogenous H_2O_2 levels

(A) H_2O_2 calibration curve using Pierce™ Quantitative Peroxide Assay Kit. (B) Calculated H_2O_2 concentration in each cell line

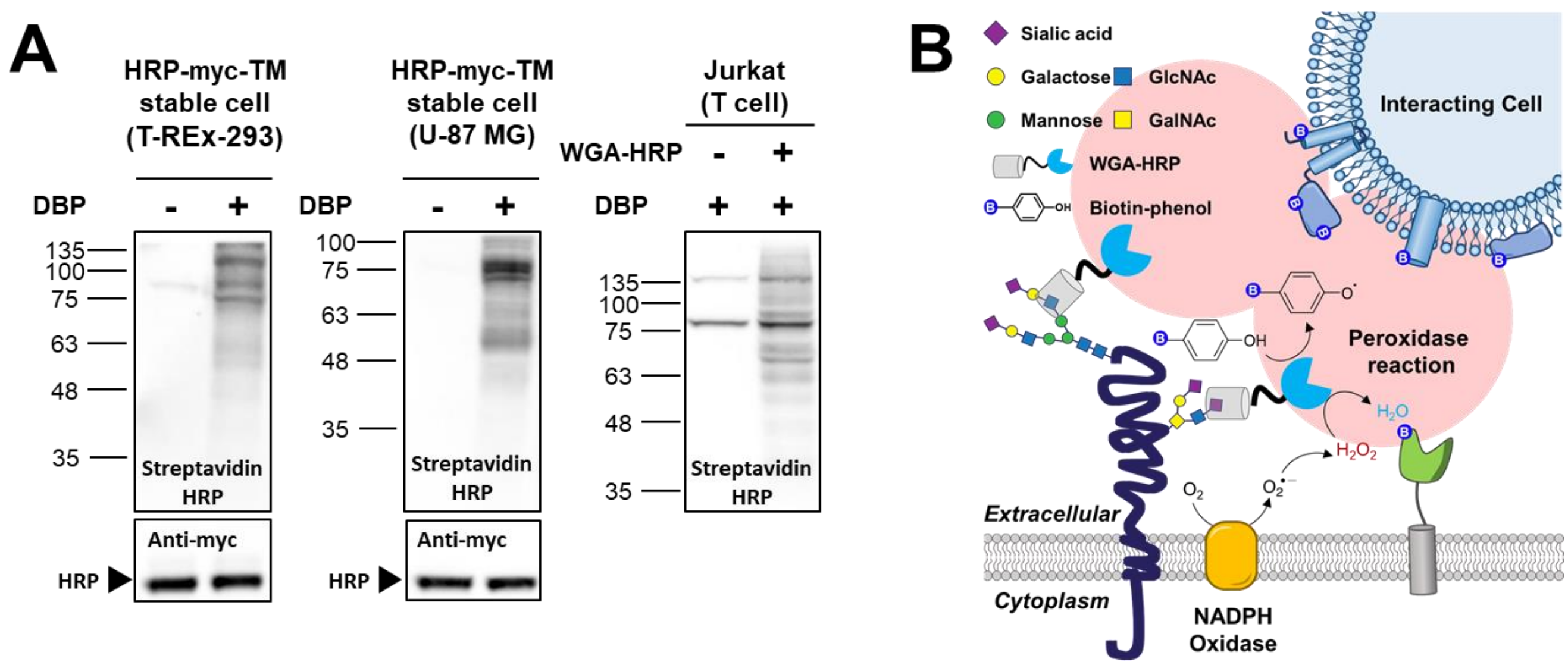

Figure S5. Labeling of various cell types via HRP

(A) Western blots of biotinylated proteins (SA-HRP) labeled by HRP-TM and WGA-HRP with or without DBP in T-REx-293, U-87MG, and Jurkat cell line. (B) WGA-HRP cell surface glycan binding and phenol probe labeling scheme. HRP-TM stably expressing T-REx-293 and U-87MG cells and WGA-HRP coated Jurkat cells were used for labeling. Labeling condition: 250 µM DBP, 30 min, 37°C.

A

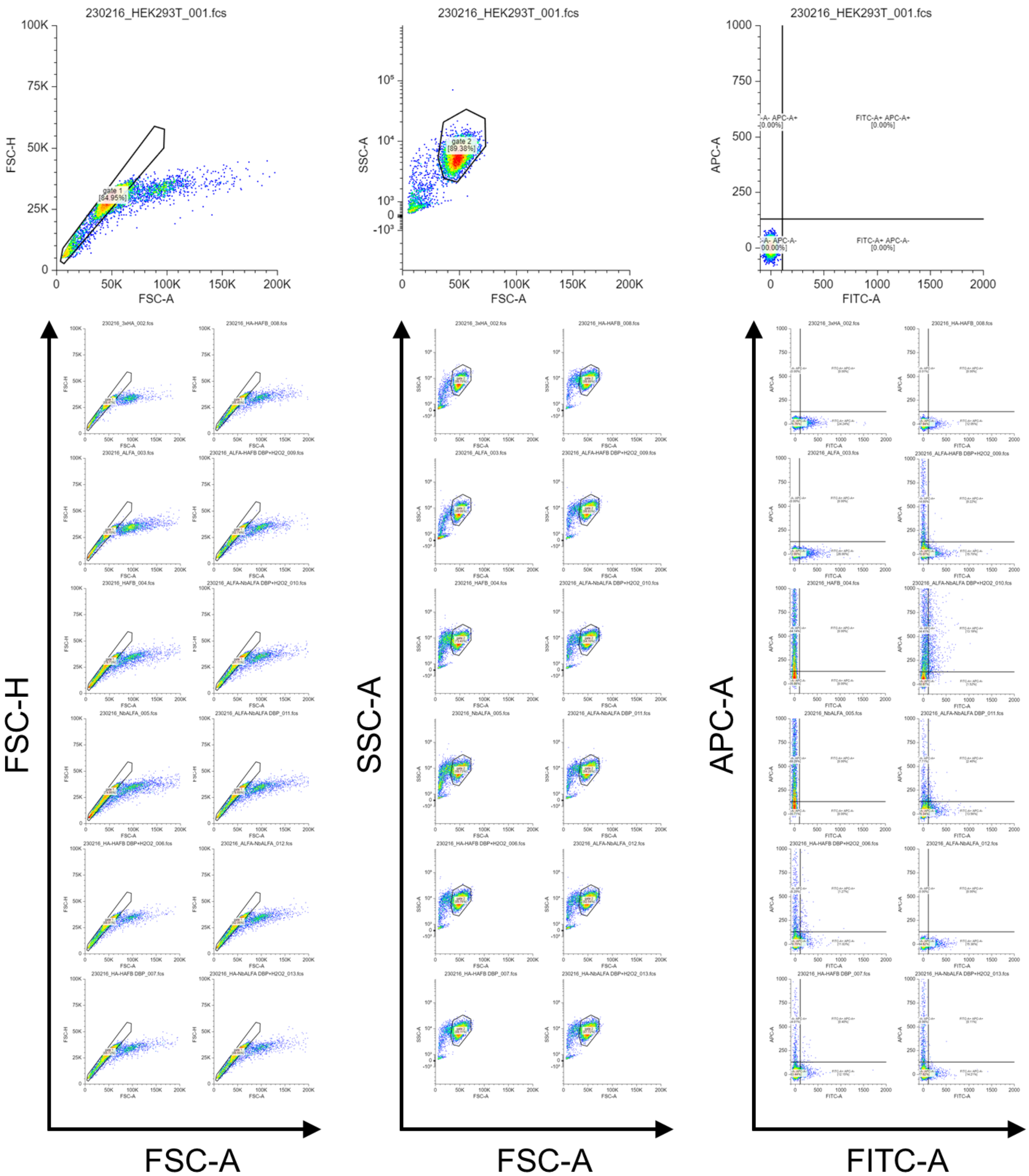
230216_HEK293T_001.fcs
FSC-H
FSC-A
gate 1
[64.95%]
SSC-A
gate 2
[89.38%]
APC-A
FITC-A
FITC-A+ APC-A+
[0.00%]
FITC-A+ APC-A-
[0.00%]
FSC-H
FSC-A
SSC-A
FSC-A
APC-A
FITC-A

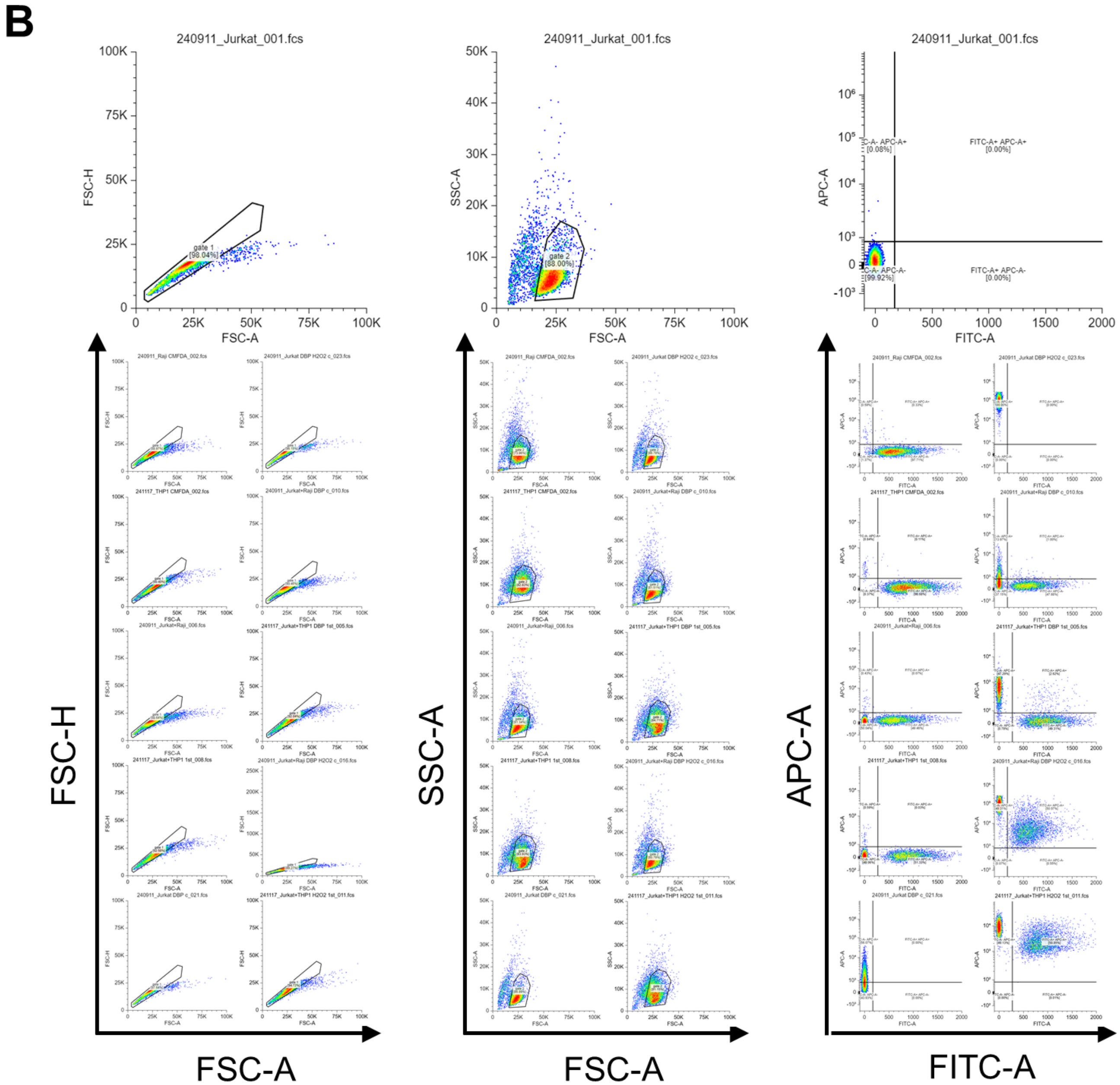

Figure S6. Gating strategy used in flow cytometry analysis

Gate 1: For extracting single cell from FSC-A and FSC-H gating, Gate 2: For extracting single population from FSC-A and SSC-A gating in gate 1 population. Quadrant: interaction analysis in gate 2 population. A is T-REx-293 cell line experiment and B is immune cell line experiment.

A

	Experiment	GFP+ (transfected) (%)			% of biotin labelled cells (SA+/Total)
		SA+ (Q2)	SA– (Q4)	Total (Q2+Q4)	
**(i)**	**Cell A + Cell a, - H_2O_2**	0.40	12.15	12.55	**3.19**
**(ii)**	**Cell A + Cell a, + H_2O_2**	1.27	11.83	13.10	**9.69**
**(iii)**	**Cell A + Cell b, + H_2O_2**	0.22	15.70	15.92	**1.38**
**(iv)**	**Cell B + Cell b, - H_2O_2**	2.40	13.56	15.96	**15.04**
**(v)**	**Cell B + Cell b, + H_2O_2**	13.19	12.52	14.71	**89.67**
**(vi)**	**Cell B + Cell a, + H_2O_2**	0.11	14.21	14.32	**0.77**

B

	Experiment	CMFDA stained (%)			% of biotin labelled cells (SA+/Total)
		SA+ (Q2)	SA– (Q4)	Total (Q2+Q4)	
**(i)**	**T cell + B cell, +DBP, -H_2O_2**	1.00	47.88	48.88	**2.05**
**(ii)**	**T cell + B cell, +DBP, +H_2O_2**	50.07	0.55	50.62	**98.91**
**(iii)**	**T cell + B cell, -DBP, -H_2O_2**	0.07	49.46	49.53	**0.14**
**(iv)**	**T cell + Monocyte, +DBP, -H_2O_2**	2.62	49.31	51.92	**5.04**
**(v)**	**T cell + Monocyte, +DBP, +H_2O_2**	50.85	0.01	50.86	**99.98**
**(vi)**	**T cell + Monocyte, -DBP, -H_2O_2**	0.03	51.33	51.36	**0.06**

Figure S7. Calculation of labeling efficiency in interacting cells

(A) GFP positive cells represent successfully transfected 3xHA-GFP-TM (cell a) or ALFA-GFP-TM (cell b). Among GFP positive cell populations, streptavidin positive cell represents interacting proximal cell. Percentage (%) of biotin labelled cells were calculated by dividing both GFP and Streptavidin (SA) positive cell population by total GFP positive population (Q2/ (Q2+Q4)).

(B) GFP positive cells represent B cell (Raji), monocyte (THP-1). Among GFP positive cell populations, streptavidin positive cell represents T cell interacting proximal cell. Percentage (%) of biotin labelled cells were calculated by dividing both GFP and Streptavidin (SA) positive cell population by total GFP positive population (Q2/ (Q2+Q4)).

A

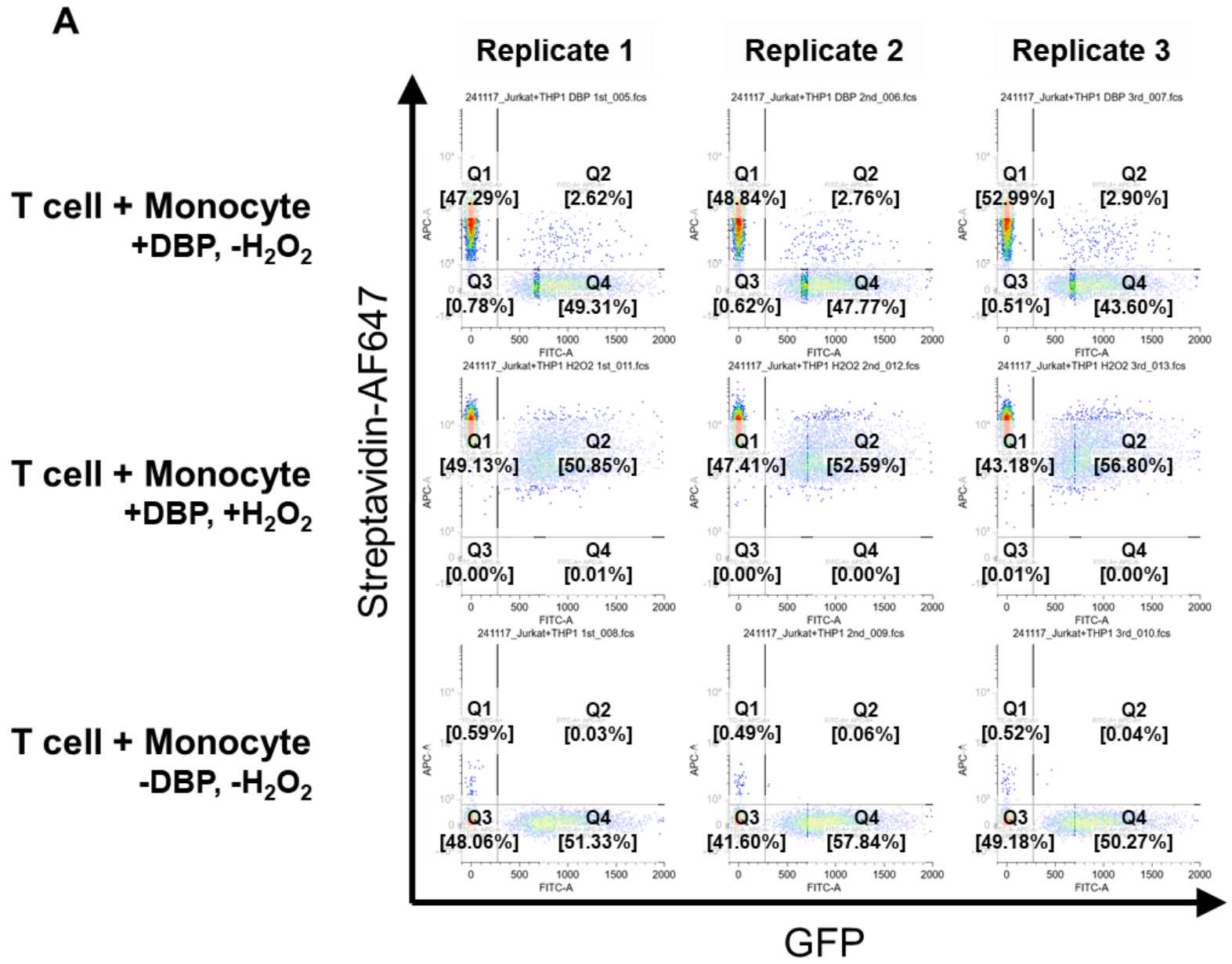

B

	Experiment	CMFDA stained (%)			% of biotin labelled cells (SA+/Total)
		SA+ (Q2)	SA– (Q4)	Total (Q2+Q4)	
1st	T cell + Monocyte, +DBP, -H_2O_2	2.62	49.31	51.93	5.05
2nd	T cell + Monocyte, +DBP, -H_2O_2	2.76	47.77	50.53	5.46
3rd	T cell + Monocyte, +DBP, -H_2O_2	2.9	43.6	46.5	6.24
1st	T cell + Monocyte, -DBP, -H_2O_2	0.03	51.33	51.36	0.06
2nd	T cell + Monocyte, -DBP, -H_2O_2	0.06	57.84	57.9	0.1
3rd	T cell + Monocyte, -DBP, -H_2O_2	0.04	50.27	50.31	0.08

C

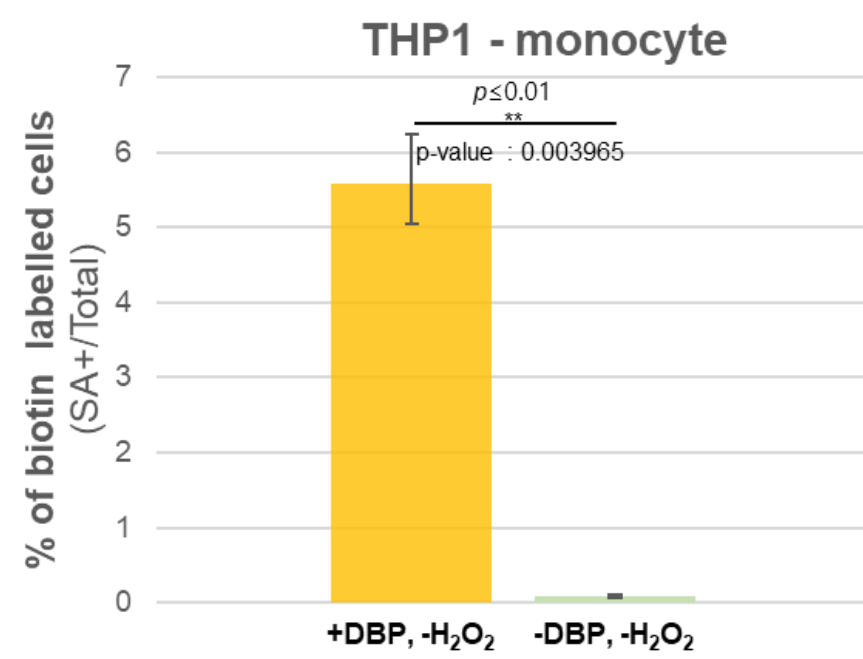

Figure S8. Triplicate experiments (T cell – monocyte cell-cell interaction) and calculated p-values
(A) Triplicate experiments of T cell – monocyte cell-cell interaction. (B) Calculation of labeling efficiency in T cell – monocyte in triplicate experiments. (C) Calculation of p-value to compare the results with and without DBP treatment.
